# Instrumental or Physical-Exercise Rehabilitation of Balance Improves Both Balance and Gait in Parkinson's Disease

**DOI:** 10.1155/2018/5614242

**Published:** 2018-03-07

**Authors:** Marica Giardini, Antonio Nardone, Marco Godi, Simone Guglielmetti, Ilaria Arcolin, Fabrizio Pisano, Marco Schieppati

**Affiliations:** ^1^Department of Translational Medicine, University of Eastern Piedmont, Novara, Italy; ^2^Centro Studi Attività Motorie, ICS Maugeri SPA SB, Institute of Pavia, IRCCS, Pavia, Italy; ^3^Neurorehabilitation and Spinal Units, ICS Maugeri SPA SB, Institute of Pavia, IRCCS, Pavia, Italy; ^4^Department of Clinical, Surgical, Diagnostic and Pediatric Sciences, University of Pavia, Pavia, Italy; ^5^Posture and Movement Laboratory, Division of Physical Medicine and Rehabilitation, ICS Maugeri SPA SB, Institute of Veruno, IRCCS, Veruno, Italy; ^6^Neurorehabilitation Division, ICS Maugeri SPA SB, Institute of Veruno, IRCCS, Veruno, Italy; ^7^Department of Exercise and Sport Science, LUNEX International University of Health, Exercise and Sports, Differdange, Luxembourg

## Abstract

We hypothesised that rehabilitation specifically addressing balance in Parkinson's disease patients might improve not only balance but locomotion as well. Two balance-training protocols (standing on a moving platform and traditional balance exercises) were assessed by assigning patients to two groups (Platform, *n* = 15, and Exercises, *n* = 17). The platform moved periodically in the anteroposterior, laterolateral, and oblique direction, with and without vision in different trials. Balance exercises were based on the Otago Exercise Program. Both platform and exercise sessions were administered from easy to difficult. Outcome measures were (a) balancing behaviour, assessed by both Index of Stability (IS) on platform and Mini-BESTest, and (b) gait, assessed by both baropodometry and Timed Up and Go (TUG) test. Falls Efficacy Scale-International (FES-I) and Parkinson's Disease Questionnaire (PDQ-8) were administered. Both groups exhibited better balance control, as assessed both by IS and by Mini-BESTest. Gait speed at baropodometry also improved in both groups, while TUG was less sensitive to improvement. Scores of FES-I and PDQ-8 showed a marginal improvement. A four-week treatment featuring no gait training but focused on challenging balance tasks produces considerable gait enhancement in mildly to moderately affected patients. Walking problems in PD depend on postural instability and are successfully relieved by appropriate balance rehabilitation. This trial is registered with ClinicalTrials.gov NCT03314597.

## 1. Introduction

Exercise is the foundation of physical rehabilitation in patients with movement disorders of various nature [1]. Increases in levels of brain-derived neurotrophic factor and brain plasticity would be accountable for this effect [2, 3]. However, much is to be desired regarding the characterization of type and dosage of the exercise to be administered to patients affected by movement disorders. Treatment for patients with Parkinson's disease (PD) is no exception [4, 5]. In their review, Tomlinson et al. [5] addressed different physiotherapy techniques but observed that evidence is insufficient for favouring one intervention over another. In the case of patients with PD, a further conundrum is represented by their high risk of falling, by their balance and gait conditions (and by the unpredictable interactions between the two functions) [6], by their reduced limits of stability [7] and biased representation of verticality [8, 9], and by their abnormal excitability of the long-loop reflexes subserving postural responses [10, 11]. In addition, they have different needs of rehabilitation treatment depending on the stage of disease and medication [12]. These conditions require adapted intervention [13, 14].

Treadmill training is a relatively popular type of exercise, because impaired locomotion is one of the most annoying signs of the disease. This training is easy to administer, can be repeated regularly, and can be practiced at home at the end of the inpatient period with relatively little cost. A recent Cochrane survey, based on data with an overall moderate quality of evidence, confirmed a mild improvement by treadmill training of gait speed and stride length [15]. A conjecture about the limited success of treadmill training is that locomotion also requires adequate balance control in addition to effective rhythmic activation of the hind limb muscles [16–18]. In this line, it is noteworthy that cycle ergometer training improves gait as much as treadmill training in PD patients [19]. Yet, balance control may be less critical during treadmill walking (or cycling) than during overground walking, since the former reduces variability in the gait parameters [20], requiring less cycle-to-cycle correction.

One might envisage that selective training of locomotion by treadmill would not be enough for comprehensive rehabilitation of walking but that optimal success could be achieved when balance is also trained [21]. Or, perhaps, even balance training alone could improve locomotion as much as treadmill training does [22]. The importance of training balance in connection with rehabilitation aimed at improving gait is easily stressed by considering the complex motor behaviour underpinning more challenging conditions than linear walking, as walking-and-turning, where the turn-related changes in feet, trunk, and head movements are integral part of the kinematics of the steering body [23–28]. It is no wonder that freezing of gait and increased risk of falling [29] is associated with abnormal bilateral coordination and turning. Hence, the present investigation somehow diverges from the theory of the task-specific training [30] but considers instead the relevance for locomotion enhancement of training balance control, by hypothesising that specific balance rehabilitation might be sufficient for gait improvement.

Here, we trained patients with PD with two different treatments, both specifically addressing balance. A platform onto which subjects stood moved in the anteroposterior, laterolateral, and diagonal direction in the horizontal plane. This platform protocol challenges both the anticipatory and the reactive capacities to the ongoing postural perturbations, thereby training dynamic balance control, aiming at the balance problems encountered during everyday activity. This protocol was based on a simpler moving-platform protocol that had been previously exploited for testing and enhancing balance capacities in patients with PD and with vestibular deficit [31–33]. The outcome of the platform treatment was compared to that obtained in another group of matched PD patients by standardised and validated exercises aimed at training balance and dynamic balance [34]. Of note, these exercises contained no dynamic component (i.e., gait-related exercises) of balance training, contrary to [35]. Both treatments (platform and exercises) were tailored to the patient individual capacities, and their difficulty gradually increased all along the duration of the treatment [35]. We estimated any improvement in balance control both by indexes of dynamic stability during a balance perturbation trial on the mobile platform and by clinical scores assessing balance control. Gait improvement was evaluated both instrumentally and by a functional clinical test.

## 2. Methods

### 2.1. Participants

Thirty-eight patients with mild to moderate idiopathic Parkinson's disease (PD) (Hoehn–Yahr stage between 1.5 and 3) were recruited from the local association of Parkinson's disease patients and from our laboratory database. All patients had a diagnosis of idiopathic PD based on defined criteria [36], and all were on stable dopaminergic medication (see Table 1). They did not change their pharmacological therapy during the study. No patient had orthopaedic conditions restricting exercise or had deep brain stimulation surgery, or showed evidence of cognitive dysfunction according to the Mini-Mental State Examination (MMSE) (Table 1). All patients could walk independently. From the patients initially screened, six were excluded according to the above criteria. Twenty-one men and eleven women finally participated in the study. The Internal Advisory Board of the Institute of Veruno approved the protocol (approval number 905 CEC), and informed consent was obtained from all the patients.

Patients were randomly assigned to two different groups of training: balance exercise training (PD-E, *n* = 17) and mobile platform training (PD-P, *n* = 15). The method of sequence generation relied on a computerized random number generator. All patients were naive to the experimental procedure, and all succeeded in performing the tasks. We used the motor section (III) of the UPDRS [37], composed of 14 items, to assess specific disorders such as bradykinesia, rigidity and tremor, balance, and functional mobility [38]. Table 1 provides the patients' characteristics for the two groups.

### 2.2. Rehabilitation Program

Each of the ten sessions was composed of 45 minutes of balance exercises (PD-E) or mobile platform (PD-P) training, each treatment being followed by a 15 min final phase of lower limb stretching, performed with the assistance of a physiotherapist. Sessions were repeated two or three times a week, with at least one rest day between one session and the next, over four successive weeks. Each patient was treated on-phase, at the same time of the day across sessions.

#### 2.2.1. Balance Exercises (17 Patients)

**Figure 1:**
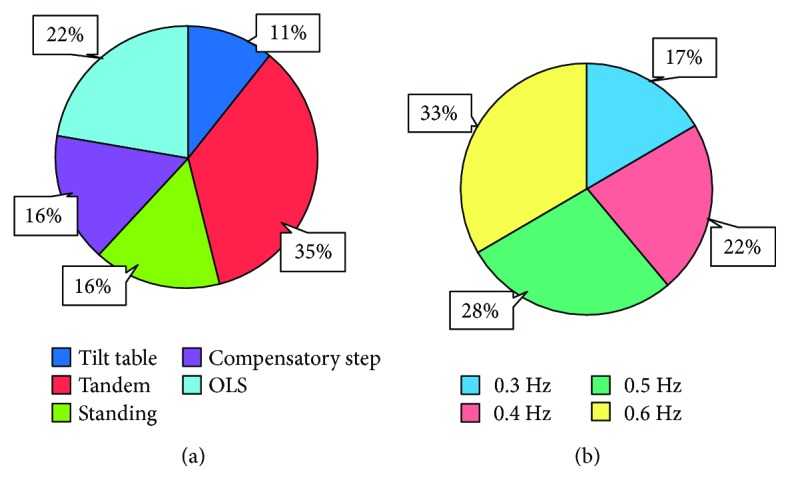
(a) Distribution of the exercise subtypes in % of the total duration of the balance training sessions. The data originate from of all patients and all sessions collapsed. OLS: one leg stance. (b) Distribution of the platform perturbation subtypes in % of the total duration of the platform training sessions, all patients, and all sessions collapsed. Each patient was trained with from easy to difficult conditions.

Patients in the PD-E group received a personalized exercise program developed by an expert physiotherapist. There was no predefined duration for each item of the set of exercises, but all patients underwent an overall 45 min period training per day according to the same schedule. This schedule (see Table 2) was based on the Otago Exercise Program [34] and practice guidelines for the treatment of Parkinson's disease [39, 40]. Patients did not wear shoes during the balance training. The exercises were performed without upper-limb support and with the supervision of a physiotherapist. This took note of the exercises performed and adjusted the progressive increase in the exercises' difficulty. In each session, each patient performed exercises in an order from easy to difficult, as depicted in Figure 1(a), based on subject-ability and item-difficulty maps of the Mini-BESTest, according to [41].

#### 2.2.2. Mobile Platform (15 Patients)

Patients (PD-P) entered the mobile platform and put on a security harness (no weight unloading), which they wore during the entire training session. Their arms were free to move, but they were asked not to reach out for support. Each patient underwent 45 minutes of training (resting periods included), in which from 6 to 8 perturbation patterns were administered, each one lasting about 4 minutes. In order to improve balance control in different directions, in separate trials, subjects stood on the platform with different whole-body orientation with respect to the platform direction of motion. During training, the platform moved in the anteroposterior, laterolateral, and diagonal (45 deg) direction with respect to the body. The periodic platform displacement was 10 cm, regardless of the frequency, which could range from 0.3 to 0.6 Hz. Patients stood with eyes open and closed and feet together or 20 cm apart depending on the perturbation subtype. There was no predefined duration for each subtype of platform perturbation, but all patients were treated according to the same progressive schedule, from easy to difficult, based on the capacity of the patient to withstand the platform perturbation configuration. The actual mean distribution and duration of the sessions is depicted in Figure 1(b).

#### 2.2.3. Stretching (All Patients)

All patients of both groups underwent a stretching exercise program as well, as recommended by several guidelines [39, 40]. The stretching program included three bouts of lower-limb muscle stretching: quadriceps, hamstring, and calf, bilaterally. Stretching was administered with the aid of the physiotherapist. It lasted about 15 minutes and was performed at the end of each session of balancing exercises or platform training.

### 2.3. Assessment Procedure

At baseline assessment, we recorded the patients' clinical characteristics (gender, age, disease duration, body weight, and height) and disability (Mini-Mental State Examination, Hoehn–Yahr staging of Parkinson's Disease, and the motor section of the Unified Parkinson's Disease Rating Scale) (see Table 1). Data collection both at baseline (T1) and at the end of training (T2, the day following the last training session) contained physical tests of balance and gait performance and scores of self-reported questionnaires about fear of falling and impact of PD on quality of life (as detailed below). All evaluations were collected by a physical therapist blinded regarding the allocation of the patients. All participants were assessed during the on-phase and at the same time of the day at T1 and T2.

#### 2.3.1. Balance Outcome Measures

These were balance behaviour indexes, assessed by (1) the dynamic balance test on the mobile platform and (2) the Mini-BESTest.

*(1) Assessment of Balancing Behaviour by Sinusoidal Translation of the Supporting Platform*. This dynamic-balance test is a sensitive tool for detecting instability in PD [42, 43]. The subjects stood upright on the platform that moved continuously 10 cm forward and backward on the horizontal plane at a frequency of sinusoidal translation of 0.4 Hz. The entire test comprised 60 cycles of motion, lasting 2 and a half min. All subjects were blindfolded, their sagittal axis coplanar with the direction of platform movement. Subjects wore a security harness and listened to music through noise-reducing earphones to mask the faint sound produced by the platform mechanism. A physiotherapist stood by the side to support the patient in case of balance loss. Body movements were recorded by detection of 3 reflective markers placed on the tragus (head), greater trochanter (hip), and lateral malleolus (invariable with respect to the moving platform). The instantaneous markers' position was recorded by means of a stereometric device (four cameras, Vicon 460, Oxford Metrics, UK) at a sampling frequency of 120 Hz. Displacements of markers were automatically interpolated and reproduced off-line by the motion analysis software.

We noted the number of cycles completed by each patient, both at baseline evaluation (T1) and at the end of the 10 training sessions (T2). Also, as an index of the average extent of back and forth displacement of the body segments in the sagittal plane (Index of Stability, IS), the standard deviation (SD) of head and hip markers' traces along the anteroposterior axis over time was computed [33, 44]. The latter is influenced both by the periodic peak-to-peak body displacements directly linked to the platform movement and by any other body displacement, not openly connected with the platform oscillation pattern. For comparison, the IS of the trace of the malleolus marker gave the reference value for the platform movement. Since not all patients performed the entire number of cycles at baseline, the calculation of the mean value of the IS was made on the cycles actually executed by patients.

*(2) Assessment of Balance by Clinical Evaluation*. The Mini-Balance Evaluation Systems Test (Mini-BESTest) is a 14-item balance scale that takes 15 min to administer. It specifically addresses dynamic equilibrium and is highly reliable [41]. Each item is scored on a 3-level ordinal scale from 0 to 2, with 2 representing no impairment and 0 representing severe impairment of balance. The total score ranges from 0 to 28. The Mini-BESTest has shown a high interrater and test-retest reliability for people with balance disorders [45] and patients with PD [46].

#### 2.3.2. Gait Outcome Measures

These were collected by (1) baropodometry and (2) the TUG test.

*(1) Assessment of Gait Performance by Baropodometry*. An electronic walkway (GAITRite®, CIR Systems, Sparta, NJ, USA) returned the baropodometric gait variables. The walkway is 460 cm long, has an area of pressure sensors of 366 cm × 61 cm containing 13,824 active sensors, and has a sampling frequency of 80 Hz. The GAITRite system has validity and test-retest reliability in patients with PD [47]. Patients were instructed to walk at their usual velocity. They began walking 2 m before the walkway and continued for 2 m past the end, in order to eliminate acceleration and deceleration events from the acquisition. After one familiarizing trial, the data from four successive trials were recorded. Gait speed, step length, and cadence were averaged over the four trials.

*(2) Assessment of Timed Up and Go Test (TUG)*. To evaluate gait in a functional situation of daily living, we used the TUG test. This is a functional measure in which subjects stand up from a chair, walk past a horizontal line marked with tape on the floor at 3 m from the start, turn around, walk back, and sit down at their comfortable pace [48]. TUG duration greater than 16 s indicates an increased risk of falls in patients with PD [49]. The test has demonstrated an excellent test-retest and interrater reliability in PD [50]. Three trials were performed, timed with a stopwatch, and the results obtained from the last two trials were averaged.

#### 2.3.3. Secondary Outcome Measures

*(1) Fear of Falling*. In order to evaluate fear of falling, all patients filled the Falls Efficacy Scale-International (FES-I). It is a self-report questionnaire developed for use in elderly populations to assess fear of falling [51]. A series of 16 questions assesses the respondent's fear of falling for a range of ADLs. Each question was rated on a four-point scale from 1 (“not at all concerned” about falls) to 4 (“very concerned”).

*(2) Impact of PD on Quality of Life*. The Parkinson's Disease Questionnaire (PDQ-8) is an 8-item self-report questionnaire derived from its parent questionnaire, the PDQ-39 [52]. It exhibits appropriate levels of reliability, validity, and responsiveness [53]. Each item was rated using a five-point scale, corresponding to the frequency with which symptoms occur (from “never” to “always”). Total score ranges from 0 to 32. A higher total score reflects a lower health-related quality of life. All patients filled both subjective questionnaires with the aid of a physiotherapist blinded regarding the allocation of the patients. No problems were encountered in correctly understanding and answering the questions.

### 2.4. Statistical Analysis

Seventeen patients were allocated in the PD-E (exercise) group and fifteen patients in the PD-P (platform) group. Sample size was chosen based on data from two previous studies performed in our laboratory employing the continuous platform perturbation as a means of training balance [31, 33]. The prospective power calculation had shown that a sample size of 15 would have 80% power to detect a mean difference in the Index of Stability of head displacement of 10 mm using a one-sided paired Student's *t*-test with alpha = 0.05.

For all recorded variables, a test for normality (Shapiro–Wilk) was performed prior to statistical comparison of the differences. To detect differences between the clinical characteristics of the two groups at T1, nonpaired Student's *t*-tests were performed for age, disease duration, body weight, and height. The Indexes of Stability (IS) of the head, hip, and malleolus in the mobile-platform assessment, the TUG duration, and the gait characteristics (speed, cadence, and step length) were normally distributed. These variables were compared between the groups at T1 by the nonpaired Student's *t*-test. The Mann–Whitney *U* test was used for MMSE, Hoehn–Yahr, and UPDRS scores.

The pre- to postrehabilitation differences of the normally distributed variables were assessed by repeated-measure ANOVA, with the groups (PD-E and PD-P) as independent factors. When ANOVA gave a significant result (*p* < 0.05), the post hoc Tukey's test was used to assess significant differences between the variables evaluated for each group.

The distribution of the number of cycles in the dynamic test proved to be nonnormal by Shapiro–Wilk test. For this reason, we used the Wilcoxon signed rank test to compare total number of cycles between pre- and post-balance treatments within each group. The same was done for the total scores of the ordinal variables (Mini-BESTest, FES-I, and PDQ-8). To assess the difference in these variables between the two patient groups at baseline and after rehabilitation, the Mann–Whitney *U* test was run.

Regression analysis was used for estimating relationships among variables, with a focus on the relationship between the value of a variable of interest posttreatment and pretreatment (considered as predictor). This analysis was made for speed and TUG duration. It was also applied to assess the degree of improvement as a function of the medication dosage.

For Mini-BESTest and gait speed, the response rate was the percentage of patients that improved after treatment, estimated by using the minimal detectable change (MDC). Cut-off for determining improvement was based on the values reported in [45] for the Mini-BESTest and [15] for gait. Differences in response rate between the groups (PD-E versus PD-P) were analyzed using Fisher's exact test (2-tailed).

Results are reported in the text and figures as mean ± SD. Statistical analysis was performed using Statistica (StatSoft Inc., Tulsa, OK, USA).

## 3. Results

### 3.1. Participant Selection

**Figure 2:**
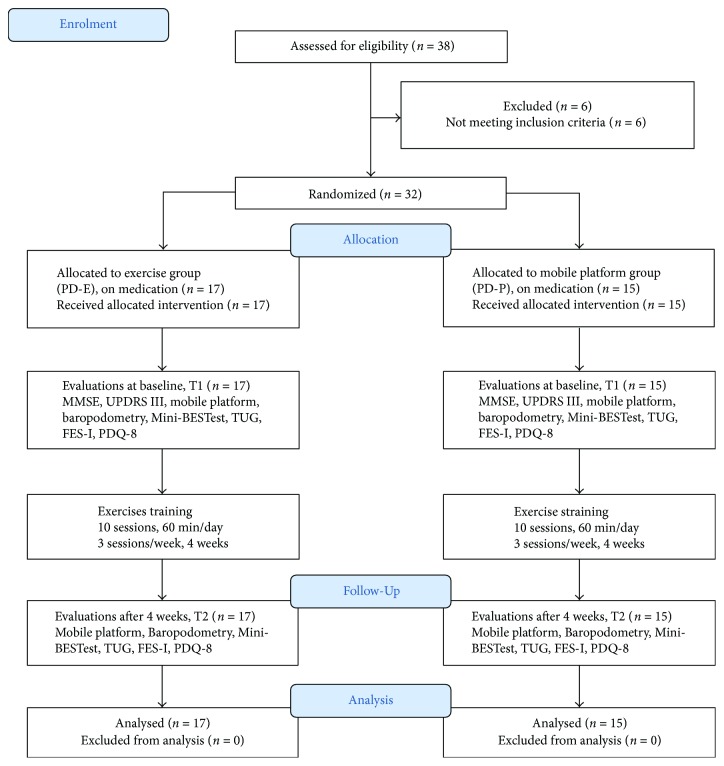
Flowchart for participant inclusion, allocation, evaluations, intervention, and analysis. Abbreviations: MMSE: Mini-Mental State Examination; UPDRS III: Unified Parkinson's Disease Rating Scale; TUG: Timed Up and Go Test; FES-I: Falls Efficacy Scale-International; PDQ-8: Parkinson's Disease Questionnaire, 8 items.

Figure 2 is a CONSORT flow diagram [54] showing the trial profile. No dropouts were recorded during the treatment, and all subjects completed the rehabilitative protocols.

### 3.2. Characteristics of the Patients

Table 1 shows the clinical characteristics of the patients. Thirteen men and four women underwent the balance exercise training (PD-E), while eight men and seven women underwent the mobile platform training (PD-P). The difference in the number of men and women between the two groups was not significant (*p* = 0.17, chi-squared test). There were no differences between the groups in age (*p* = 0.56), disease duration (*p* = 0.92), MMSE (*p* = 0.89), Hoehn–Yahr stage (*p* = 0.33), and UPDRS motor score (*p* = 0.28).

### 3.3. Effects of the Rehabilitation Treatments (PD-P and PD-E Groups)

#### 3.3.1. Improved Balancing Behaviour as Assessed by the Sinusoidal Translation of the Supporting Platform and by the Mini-BESTest

**Figure 3:**
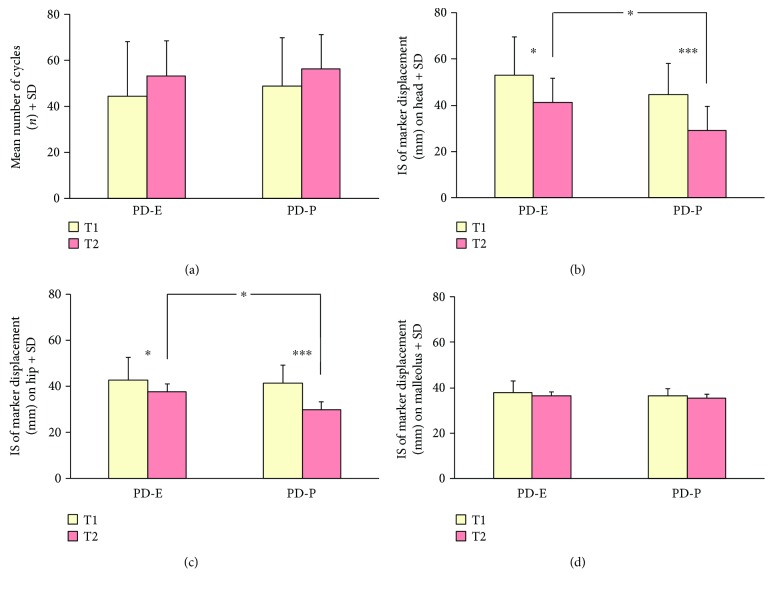
Training effects on body stabilization assessed by the moving-platform test in the two groups of patients (PD-E and PD-P) at baseline (T1, yellow columns) and after treatment (T2, pink columns). All subjects were tested at 0.4 Hz perturbation frequency, eyes closed. At T2, patients endured longer periods on the platform than at T1 (a). Head (b) and hip (c) displacement (Index of Stability (IS)) improved significantly after both platform and exercise training, indicating a decrease in body segment oscillation. IS at T2 was better in the PD-P than in the PE-E group for both the head and hip. (d) shows that feet position on the platform was substantially unvarying for patients in both groups. Asterisks (^∗^*p* < 0.05; ^∗∗∗^*p* < 0.0005) indicate differences (T1, T2) within groups and between groups at T2.

*(1) Moving Platform*.  Figure 3(a) shows the effects of both treatments on the dynamic balance test with the mobile platform. At the baseline evaluation (T1), only 10 patients in each group were able to complete the test (up to 60 cycles). No difference was present in the mean number of cycles between the two groups at T1 (*p* = 0.59). After training (T2), more patients completed the test (13/17 PD-E and 14/15 PD-P) and each patient endured more perturbation cycles. No difference was present in the mean number of cycles between the two groups at T2 (*p* = 0.47). In spite of enduring longer perturbation periods, no significant difference was found in the mean number of cycles between T2 and T1 within each group, likely because of the large variability across patients (*p* = 0.35 and *p* = 0.28, for PD-E and PD-P, resp.).

Figures 3(b) and 3(c) show the mean Indexes of Stability (IS) of the head and hip at T1 and the changes at T2 in the two training groups. A decrease in the IS denotes better segment stabilization in space. Two-way ANOVA on the IS of the head showed a difference between the groups (*F*(1,30) = 7.60, *p* < 0.05). There was an overall positive effect of training (*F*(1,30) = 33.24, *p* < 0.0005). The interaction between group and treatment did not reach significance (*F*(1,30) = 2.53, *p* = 0.10). The post hoc test showed no significant difference between the two groups at T1 (*p* = 0.35). At T2, the IS of the PD-P was smaller than that of the PD-E group (*p* < 0.05) (Figure 3(b)). As to the mean IS of the hip marker, two-way ANOVA showed again a difference between the groups (*F*(1,30) = 4.21, *p* < 0.05) and a significant effect of training (*F*(1,30) = 55.75, *p* < 0.0005). The interaction was significant (*F*(1,30) = 9.55, *p* < 0.005). Post hoc analysis showed no difference at T1 (*p* = 0.94). At T2, the IS of hip marker improved more in the PD-P than in the PD-E group (*p* < 0.05) (Figure 3(c)). As a control, we recorded also the IS for the malleolus marker (Figure 3(d)). Two-way ANOVA showed no difference between the groups (*F*(1,30) = 2.18, *p* = 0.15). There was a small effect of training (*F*(1,30) = 3.79, *p* < 0.05), connected to the reduction at T2 of the small displacements of the feet during the perturbation cycles, which was instead present at T1. The training-group interaction was not significant (*F*(1,30) = 0.002, *p* = 0.97).

**Figure 4:**
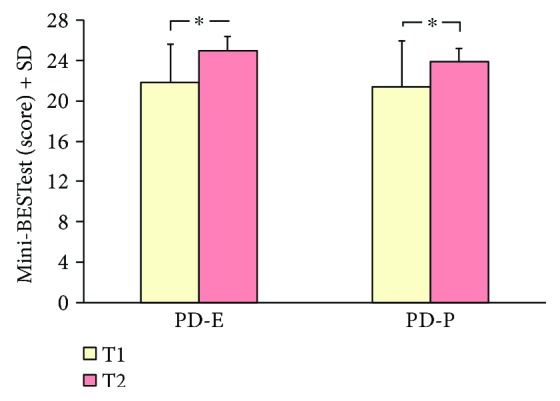
Training effect on balance, measured by the total score of the Mini-BESTest. Yellow columns represent pretraining and pink columns posttraining evaluation. A significant difference was found between T1 and T2 within each group (Wilcoxon test; ^∗^*p* < 0.05).

*(2) Mini-BESTest*.  Figure 4 shows the average score for the Mini-BESTest at T1 and T2 in the two patient groups. At baseline, both groups showed no difference in the scores (Mann–Whitney *U* test, *p* = 0.91). After training, scores increased significantly in both groups (Wilcoxon test, *p* < 0.01). No significant difference (Mann–Whitney *U* test, *p* = 0.18) was found between the groups after training.

#### 3.3.2. Improved Locomotion as Assessed by Baropodometry and by the Timed Up and Go Test

**Figure 5:**
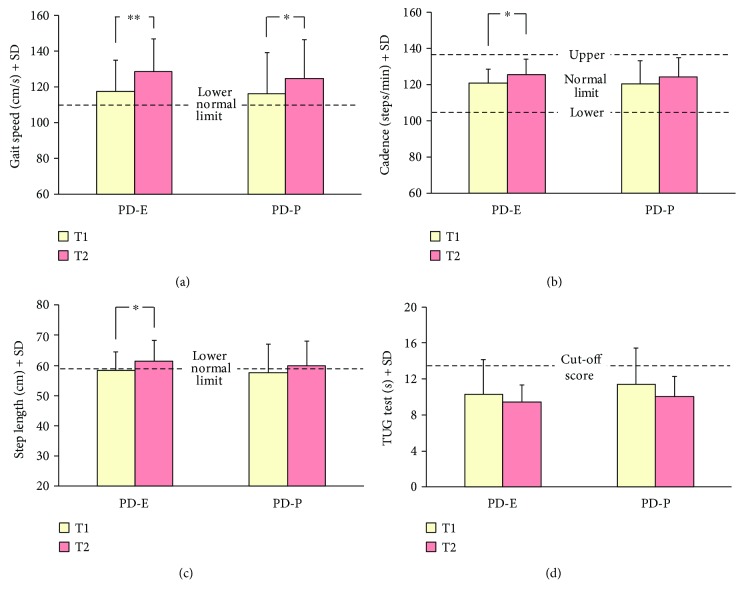
Analysis of the training effects assessed by the baropodometric and clinical measures collected in the two groups at baseline (T1, yellow columns) and after the treatment (T2, pink columns). Gait speed (a) significantly improved in both groups, while cadence (b) and step length (c) increased only slightly (significantly so in PD-E). Dashed lines indicate the limits of normality. Time to perform the TUG test (d) slightly diminished in both groups; cut-off score for fall risk is indicated by the dashed line. Asterisks (^∗^*p* < 0.05; ^∗∗^*p* < 0.005, Tukey's post hoc test) indicate differences within group. No difference was found between groups after training for any variable.

*(1) Baropodometry*. At baseline, the spatiotemporal variables of gait (speed, cadence, and step length) were not different for PD-E and PD-P. After training, gait variables improved significantly. Two-way ANOVA showed that there was an effect of training on gait speed (Figure 5(a)) (*F*(1,30) = 19.50, *p* < 0.001). No difference was found between the groups (*F*(1,30) = 0.17, *p* = 0.68). There was no interaction (*F*(1,30) = 0.29, *p* = 0.60). Post hoc analysis showed that gait speed at T1 (*p* = 0.96) and at T2 (*p* = 0.94) was similar in both groups.

As to gait cadence (Figure 5(b)), two-way ANOVA showed a significant pre- to posttraining difference (*F*(1,30) = 13.18, *p* < 0.005). Cadence slightly increased from 121.0 ± 7.5 steps/min to 125.6 ± 8.6 steps/min and from 120.5 ± 12.9 steps/min to 124.3 ± 10.6 steps/min, for PD-E and PD-P, respectively. No difference was found between the groups (*F*(1,30) = 0.07, *p* = 0.80). The interaction between groups and treatment was not significant (*F*(1,30) = 0.15, *p* = 0.70). Post hoc analysis showed no difference in cadence between the groups, either at T1 or at T2 (*p* = 0.99 and *p* = 0.98, resp.), and a small significant increment in cadence for PD-E (*p* < 0.05) but not for PD-P (*p* = 0.14).

There was a modest but significant effect of training on step length (ANOVA, *F*(1,30) = 17.16, *p* < 0.0005) (Figure 5(c)). No difference was found between the groups (*F*(1,30) = 0.20, *p* = 0.66), and the interaction was not significant (*F*(1,30) = 0.36, *p* = 0.55). Post hoc analysis showed no difference in step length between the groups, either at T1 or at T2 (post hoc test, *p* = 0.99 and *p* = 0.94, resp.), a significant increment in step length at T2 for PD-E (*p* < 0.01), and a marginal increment for PD-P (*p* = 0.06). At the end of the training, the mean values of step length slightly exceeded the lower limit of normality in both groups.

**Figure 6:**
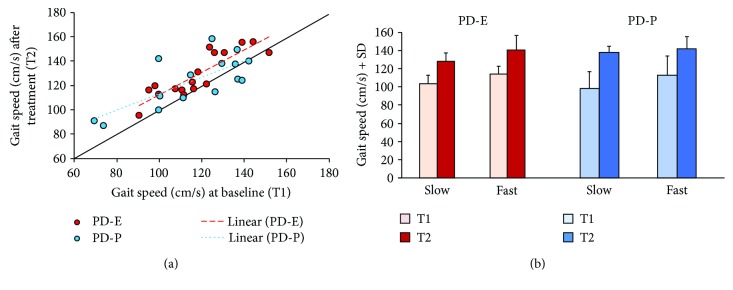
(a) This shows the correlation between gait speed pre- and posttreatment, as assessed by baropodometry. Red and blue circles represent all single subjects of the PD-E and PD-P groups, respectively. Most data points lay above the identity, indicating increased walking speed in most patients. (b) The patients with a lower gait speed at T1, belonging to both treatments groups, did not show a statistically significant disproportionate improvement after training.

Figure 6 shows the distribution of the individual data points pertaining to gait speed. Speed values at T2 are plotted against speed values at T1, for each patient of both groups. In spite of a considerable scatter of data points in both populations, the regressions were significant (*y* = 0.92*x* + 20.42, *R*^2^ = 0.75, and *p* < 0.0001 and *y* = 0.68*x* + 44.98, *R*^2^ = 0.54, and *p* < 0.001, for PD-E and PD-P, resp.). Speed increment was shown by most patients, except three in the PD-P group (below the identity line), for whom the gait speed was pretty high already at T1. The slope of the two regression lines was not significantly different from the identity line for either group (*p* = 0.56 and *p* = 0.11, for PD-E and PD-P, resp.), suggesting that the patients with lower gait speed at T1 did not exhibit a more effective improvement after either training protocol.

We have checked this by an ANOVA, separately run for the two treatments. For each treatment, the gait speeds at T1 were sorted into slow and fast subgroups. Accordingly, ANOVA showed a significant difference in speed between subgroups (PD-E, *F*(1,15) = 28.37, *p* < 0.0001; PD-P, *F*(1,13) = 25.84, *p* < 0.0001). There was a significant effect of treatment on velocity at T2 (PD-E, *F*(1,15) = 22.25, *p* < 0.0001; PD-P, *F*(1,13) = 5.29, *p* = 0.038), but there was no interaction between subgroup and velocity (PD-E, *F*(1,15) = 0.02, *p* = 0.88; PD-P, *F*(1,13) = 1.73, *p* = 0.22), indicating no extra advantage for the patients with a slow gait speed at T1, for either the exercise or the mobile platform treatment.

*(2) Timed Up and Go Test*.  Figure 5(d) shows the average changes of TUG duration for PD-E and PD-P patients at T1 and T2. The dashed line indicates the cut-off score for fall risk in TUG [54]. Two-way ANOVA showed that, both groups collapsed, training significantly decreased the mean TUG duration (*F*(1,30) = 6.68, *p* < 0.05). No difference was found between the groups (*F*(1,30) = 0.64, *p* = 0.43), and the interaction was not significant (*F*(1,30) = 0.37, *p* = 0.55). Post hoc analysis showed that TUG duration was similar in both groups both at T1 (*p* = 0.77) and at T2 (*p* = 0.96). When the effect of treatment was assessed by the post hoc test within each group, the increments did not reach significance, likely because of the limited number of patients per group (*p* = 0.48 and *p* = 0.15, for PD-E and PD-P, resp.).

**Figure 7:**
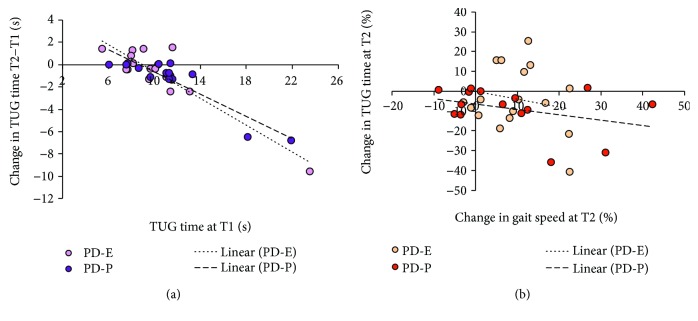
(a) The scatterplot shows the changes in TUG time (T2–T1) plotted against the TUG time at T1 for each patient of both groups. For most patients, TUG time at T1 was close to the normal values of age-matched healthy subjects. The decrease in time was limited (or absent) in most cases, except for three patients, who improved much their initial performance. (b) Percent changes in TUG time after rehabilitation were not related to the percent improvement of gait speed assessed by baropodometry.

Figure 7(a) shows that the decrement in TUG duration was remarkable just for the few subjects, the TUG time of which was long at T1, corresponding to a high risk of falling, but that no major effect was noted in the subjects having scores close to normal at T1. Both regressions were significant (*y* = −0.60*x* + 5.35, *R*^2^ = 0.82, and *p* < 0.001 and *y* = −0.50*x* + 4.29, *R*^2^ = 0.83, and *p* < 0.001, for PD-E and PD-P, resp.). There was no obvious difference between the two groups.

*(3) Correlation between Gait Velocity and Timed Up and Go Test*.  Figure 7(b) shows the correlation between the improvement in TUG scores and the increase in gait speed at baropodometry, for both groups. Both treatments produced a limited and scattered percent change in TUG scores except in a few patients. Neither regression was significant (*y* = −0.40*x* + 0.02, *R*^2^ = 0.04, and *p* = 0.45 and *y* = −0.27*x*–6.32, *R*^2^ = 0.14, and *p* < 0.17, for PD-E and PD-P, resp.).

### 3.4. Response Rate of the Two Protocols for Balance and Gait

Based on the value of the minimal detectable change (MDC) for the Mini-BESTest, published in [45] (3.5 points), the treatments improved the score in 11/17 patients of the PD-E group and in 5/15 patients of the PD-P group. Differences between groups did not reach significance (Fisher's exact test = 3.14, *p* = 0.08). A similar picture applies to gait speed, by using the MDC published in [15] (0.09 m/s). Treatments improved speed in 10/17 patients of the PD-E group and 7/15 patients in the PD-P group. The difference between the two rates did not reach significance, either (Fisher's exact test = 0.47, *p* = 0.49).

**Figure 8:**
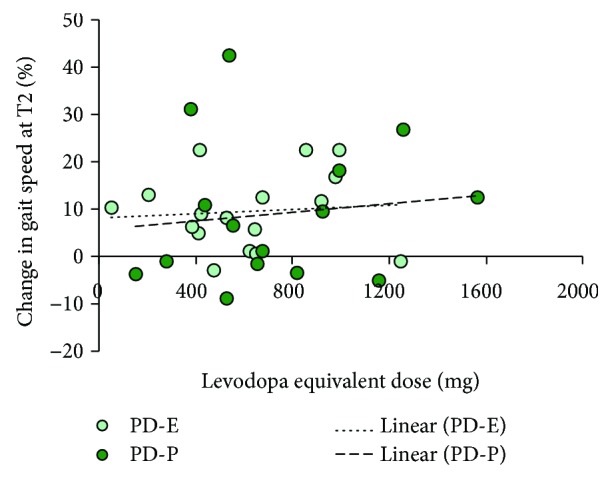
The regression lines are drawn through the data points representing the percent changes in gait speed at T2 against medication. There was no effect of total medication (expressed as levodopa equivalent dose) on changes in gait speed, in either treatment group.

Figure 8 shows the result of the attempt at evaluating any interaction between medication and the effects of the rehabilitation treatments on gait speed, in the assumption that the amount of medication might affect the outcome of the treatments.

For each patient, the percent change in gait speed is plotted as a function of the levodopa equivalent dose. Regression lines were drawn through the data points. For both groups, the lines were almost superimposed, and their slope was not significant (*y* = 0.002*x* + 8.12, *R*^2^ = 0.008, and *p* = 0.74 and *y* = 0.005*x* + 5.48, *R*^2^ = 0.0016, and *p* = 0.66, for PD-E and PD-P, resp.).

### 3.5. Scores of Self-Reported Questionnaires about Fear of Falling and Impact of PD on Quality of Life

#### 3.5.1. Fear of Falling

There was a marginal effect of training on the total score of FES-I (not shown). At baseline, the average score of FES-I was 23.5 ± 7.1 and 26.3 ± 10.7 (Mann–Whitney *U* test, *p* = 0.63) for PD-E and PD-P, respectively. After training, the average values decreased to 21.2 ± 5.4 and 24.3 ± 11.0, respectively. The reduction was marginally significant (Wilcoxon test, *p* > 0.08 for both comparisons). No significant (Mann–Whitney *U* test, *p* = 0.38) difference in the scores between the groups was found after training.

#### 3.5.2. Impact of PD on Quality of Life

At baseline, the average score of PDQ-8 was 5.9 ± 6.1 and 8.9 ± 7.1 (Mann–Whitney *U* test, *p* = 0.16), for PD-E and PD-P, respectively (not shown). After training, the average values decreased to 4.4 ± 4.7 and 6.8 ± 6.3, respectively. The reduction was marginally significant (Wilcoxon test, *p* = 0.06 for both groups). No significant (Mann–Whitney *U* test, *p* = 0.14) difference between the groups was found after training.

## 4. Discussion

The interplay between balance and gait disorders in Parkinson's disease is a matter of debate. Poor postural control is common in PD patients, is not always improved by dopaminergic drugs [55, 56], and interferes with walking [57]. In other diseases, walking problems are strongly dependent on impaired balance control as well, as in peripheral neuropathy [33], stroke [58], and cerebellar syndromes [59]. In PD, multiple abnormal neural circuits may be responsible for balance and gait problems [56]. The pars reticulata of the substantia nigra seems to play a major role in the control of dynamic balance, while other basal ganglia nuclei exert a superior control on gait [60]. Mille et al. [61, 62] addressed the integration of postural motor actions for both balance stabilization and locomotion in normal subjects and suggested that feedforward adaptation of locomotion depends on the current postural conditions. Humans have the ability to control the position of the centre of body mass on a restricted base of support in order to maintain equilibrium in both gait [63] and upright quiet stance [64] and use the vertical as a reference for the coordination for both postural responses and locomotion [8]. So, the processes for the control of posture and locomotion are likely to be interdependent at many different levels of the central nervous system and share some common principles of organization [57, 64]. On the one hand, the links between balance and gait would be compromised in PD, and this is perhaps the reason why Massion [65] found that balance and gait represent independent domains of mobility in Parkinson's disease. On the other hand, one may note that dopaminergic medications and deep brain stimulation of the subthalamic nucleus provide similar improvements in balance and gait tasks [66].

Beyond the time-honoured notion that physical activity of different nature and intensity improves activities of daily life in PD [67, 68], it is not known whether dynamic-balance rehabilitation per se can improve not only balance control but locomotion as well. This information would have both theoretical implications (locomotion is degraded in PD mainly because of poor balance control—once the latter is improved, walking becomes more manageable) and practical implications (identifications of balance rehabilitation protocols as the main intervention). Interestingly, a manoeuvre consisting in alternate rhythmic vibration of trunk paraspinal muscles during quiet stance was able to produce a cyclic mediolateral transfer of the centre of pressure [69], mimicking accompanying body progression during walking. When the same type of vibration was applied during gait, walking velocity, cadence, and stride length improved [70].

Here, we studied two populations of patients with PD on stable medication, matched for age and severity of disease. They were trained with two protocols specifically aimed at improving dynamic balance and trunk control. In no case was specific exercises for gait rehabilitation or treadmill training included. One group underwent a program based on classified physical exercises, targeting balance. Another rode a platform, which moved 10 cm back and forth sinusoidally in different directions, a treatment that challenges dynamic balance by forcing subjects to maintain equilibrium and adapt to the continuous postural perturbation [33, 44, 71–73]. Both treatments were tailored to the patients' tolerance and gradually increased in difficulty across subsequent sessions as a function of progressive improvement in patients' compliance [4, 35, 74]. Balance-training exercises are known to improve postural control in patients with Parkinson's disease [75, 76]. Instrumental rehabilitation (continuous displacement of a platform upon which subjects stood) has been successfully exploited for improving balance in vestibular [31] and neuropathic patients [33]. Both protocols instruct stabilizing responses to postural perturbations and are likely to improve trunk control [77]. They also contain part of the tasks proposed for the rehabilitation of the sense of verticality [9], which is certainly related to balance and equilibrium, and are appropriate for challenging sensorimotor integration mechanisms [78].

Protocols partly differ, though. During the exercises, the base of support and the feet position are being deliberately changed by using different materials (solid, foam), inclinations (flat, tilted), features (feet parallel, semi-tandem, tandem, and one leg stance), and intervention (push and release). During the mobile platform protocol, the feet are parallel and their position in relation to the platform does not change. The periodic platform displacement trains adaptation to balance perturbation by a mix of ankle and hip strategies of different relative weight and by learning to anticipate postural adjustments [73].

Both balance-training protocols decreased the periodic oscillations of the head and hip, as tested by means of continuous and periodic anteroposterior displacement of the support base. The PD-P group reduced head and hip oscillations slightly more than the PD-E group. In particular, the centre of mass (the hip marker) appeared to be better controlled in the patients to whom platform training was administered (PD-P). Most likely, this is connected with the PD-E patients being naïve to the platform, while the PD-P patients were already familiar with it because the treatment was based on a mobile-platform protocol and were likely less susceptible to the startling effects of the platform displacement [73, 79]. Yet, both protocols had definite positive effects on the capacity to counteract the balance perturbation test. When assessed by the Mini-BESTest, the patients belonging to both groups showed a significant and similar improvement in dynamic balance. At T1, both groups had been scored “moderate deficit,” and at T2, both were scored “mild deficit to normal” [80]. The latter is the same category within which healthy subjects in the same age range are comprised [81]. Thus, both the exercise and the platform treatments have had an effect on dynamic balance. These effects, though modest, occurred in spite of the patients assessed at T1 by the Mini-BESTest not exhibiting major balance problems.

The improvement in locomotion was hypothesised, but not necessarily expected. In both groups, walking velocity increased, suggesting that balance exercises or an instrumental protocol aimed at improving equilibrium has a positive effect on walking speed. The baropodometric findings showed that, at T1, both groups' mean step length was below the lower limit of normality. At T2, step length increased and rose above the lower limit of normality in both groups. Cadence increased to a limited extent, coherent with [15] and with the described relationship between stride length and cadence [82]. Hence, normalization of step length was helpful for increasing walking speed. During the single support phase, propulsion is achieved by slowly braking the gravity-induced fall of the body through controlled activation of the triceps surae muscle: the longer the triceps activation in the supporting leg, the farther the heel contact of the foot of the swinging leg, therefore, the higher the velocity at constant cadence [83, 84]. This phase of the gait cycle is therefore substantially a “postural” task, whereby the triceps surae and erector spinae muscles dynamically control equilibrium [77, 85, 86]. This outcome is particularly significant in PD patients, in which step length is short because of impaired capacity of counteracting gravity, leading to the swing leg to fall short of the expected distance [87].

The time to perform the TUG test also diminished concurrently with the increase in step length in both groups. However, the decrement was modest in both groups. In this connection, Podsiadlo and Richardson [48] found TUG scores of 10 s or less in healthy elderly people. In our study, the PD patients had scores close to 10 at T1. Both groups had an average Hoehn–Yahr stage of 2.3, and this value identifies a mild disease [88]. These factors would explain the unremarkable improvement in the TUG test. Schenkman et al. [89] performed the TUG in PD patients, categorized into H&Y staging, and their findings suggest that limitations in the TUG are not revealed until later in the disease progression, when fatigue and decreased muscle strength would predominate [90]. So, likely because of its relative complexity compared to straight walking, and because of a probable ceiling effect, TUG may be inferior to baropodometry for estimating the effects of treatment on walking speed.

Even though the period of training was relatively short, there was a slight tendency of reduction of the fear of falling of patients during the execution of activity of daily living, as evaluated with the FES-I scale. Again, no differences between patient groups were found. Similarly, in the PDQ-8 scale that evaluates the health-related quality of life, there was a slight tendency to reduction, without the prevalence of one approach over the other.

All in all, the findings are in keeping with the hypothesis that treatments aimed at improving the dynamic control of balance improve locomotion in PD patients. In a different group of PD patients, Arcolin et al. [19] observed an increase in gait speed after both treadmill and cycloergometer training. The effect was slightly larger than that observed here, but those patients exhibited a smaller walking velocity at T1 than the present cohort. Others reported increments in walking velocity with treadmill training [91, 92]. A recent Cochrane review showed that treadmill training improves gait speed with respect to no intervention, with an effect size of about 0.40 [15]. In our cohorts, we estimated an effect size for gait speed of about 0.55 and 0.40, for balance exercise and mobile platform groups, respectively. This indicates that both exercise and mobile platform protocols produced an increment comparable to that of the treadmill training, which is considered the standard intervention for gait rehabilitation [93]. The mobile-platform training increased gait speed very much like the exercises, in spite of both feet resting on the platform all the time—a task certainly opposite to rhythmic alternate leg movements. Conversely, mobile-platform training would have improved head-trunk-leg coordination, thereby normalizing the activity of trunk muscles, which is affected in Parkinson's disease [94]. From a practical point of view, as also assessed by the evaluation of the response rates, both treatments alike proved to be similarly effective. Naturally, the mobile-platform treatment has the benefit of being quantifiable, standardised, repeatable, and independent from the operator. Hence, a simple device producing a back-and-forth periodic displacement of the support base, with the option of modifying the frequency and direction of oscillation, seems to be appropriate for both balance *and* gait rehabilitation in Parkinson's disease.

Of note, all our patients were on medication, with the drug dosage spanning in an ample range within and across groups. On the one hand, it is unclear whether medication affects the capacity of the patients to improve in response to rehabilitation. On the other hand, the effect of levodopa on balance and gait is controversial [95, 96] or limited [97]. Therefore, it was interesting to investigate whether patients improved more or less after rehabilitation as a function of the equivalent total dose of levodopa [98]. No obvious interaction between instrumental or physical-exercise rehabilitation of balance and medication was found. Yet, based on our admittedly small sample, and on the lack of an ad hoc control group trained under medication-off condition, we would not express any strong statement in favour of an enabling effect of medication on the rehabilitation-induced improvement of walking.

All in all, we would therefore argue that dynamic-balance rehabilitation is sufficient for improving locomotion in PD patients. We would also put forward the notion that walking problems in PD depend on, or are very closely related to, balance impairment. This extends to PD the idea that walking velocity is affected by postural instability, very much as it has been suggested for cerebellar and neuropathic diseases [58, 59, 99] or patients with stroke [100] or COPD [101]. To our knowledge, this is the first study that shows improvement in gait induced by mere balance training in patients with PD. In other types of neurological disease, such as spinal injury patients, it was found that balance exercises improved gait [22, 102]. However, in both studies, subjects were administered exercises also focused on subcomponents of gait.

### 4.1. Limitations

The relatively unexceptional improvement in the spatiotemporal variables of gait, in both groups, may have been limited by the values of these variables at baseline. Patients recruited in this study had an average Hoehn–Yahr stage of just 2.3, featuring spatiotemporal variables of gait at baseline only slightly off the normal range. The effects on gait of these exclusive balance trainings are based on a small sample size and should be confirmed in a larger cohort, also including more severely affected PD patients. In this line, we would also note that our balance treatments did not overly improve the Mini-BESTest scoring, either, most likely because the patients' scores at T1 were close to those of normal subjects of the same age.

We do not know whether longer treatments (e.g., [103]) might have granted greater improvements nor are we in the position of arguing about the duration of the positive posttreatment effects, in either group. However, based on previous observations in vestibular patients treated in a similar way [31], it would not be improbable to observe a protracted improvement.

### 4.2. Conclusions

A four-week balance treatment, not containing any gait-rehabilitation exercises, is sufficient for producing considerable improvement in walking velocity in mildly to moderately affected patients with PD. Although these conclusions are based on small patient numbers, the data are in keeping with the hypothesis that balance control is paramount for locomotion in Parkinsonian patients. One could be even justified to posit that locomotion is degraded in PD *because* of poor balance control and to advocate rehabilitation of balance as a priority for enhancing gait.

## Figures and Tables

**Table 1:** Demographics, clinical details, and medication of the 32 patients with PD who participated in the study, divided into the two training groups: PD-E (exercises) and PD-P (platform). The two groups were homogeneous at baseline, as shown by Student's *t*-test and Mann–Whitney *U* test.

Group	Sex	Age (years)	Body weight (kg)	Height (cm)	Duration (years)	MMSE	Hoehn–Yahr	UPDRS motor score	Medication (mg/die)	LED (mg)
PD-E	M	70	76	160	12	27.03	3	12	LD (937.5), PR (2.1), RA (1)	1248
	M	75	83	178	4	23	2	20	PR (3.15), RA (1)	415
	M	63	75	160	4	29	2	10	LD (187.5), RA (1), RT (8)	528
	M	71	63	167	6	27	2	17	LD (375), PR (0.52), RA (0.5)	477
	W	68	62	168	11	30	2.5	20	EN (800), LD (500), RA (0.5), RO (8), RT (4)	995
	M	81	75	167	8	26.4	2	12	LD (625), PR (0.52)	677
	W	58	53	167	11	30	2	21	EN (800), LD (656.25), PR (1.05)	978
	M	66	69	166	3	27.9	2.5	11	LD (437.5), RO (24)	918
	M	66	65	172	6	27.9	2.5	21	LD (125), RA (1), RO (8)	385
	M	71	65	165	12	25.4	2.5	18	EN (400), LD (250), PR (2.1), RA (1)	643
	W	51	90	173	10	30	2	19	LD (312.5), PR (2.1), RA (1)	623
	M	70	76	180	9	30	2.5	22	AM (50), EN (600), LD (375), PR (0.52), RA (0.5)	651
	M	54	68	167	7	30	2.5	31	RA (1), RO (16)	420
	W	72	63	160	1	30	1.5	9	PR (0.52)	52
	M	69	72	170	5	26.9	2	17	LD (125), RA (1), RO (10)	425
	M	80	55	170	8	28.7	2.5	13	LD (500), PR (1.57), RA (2)	857
	M	71	82	168	1	25.3	3	24	PR (1.05), RA (1)	205
Mean		68.0	70.1	168.1	6.9	27.9	2.3	17.5		617.5
SD		8.0	9.9	5.6	3.6	2.1	0.4	5.8		307.4

PD-P	M	66	85	179	13	26.9	2.5	14	LD (125), PR (2.1), RA (1), RT (8)	675
	W	67	75	152	10	23	2.5	13	AM (200), LD (1000), PR (0.26), RA (1), RT (8)	1566
	M	79	62	170	12	23	2	20	M (150), EN (800), LD (637.5), PR (2.1), RA (0.5)	1258
	W	66	49	170	6	27	2.5	19	PR (3.41), RA (1)	441
	M	80	69	160	8	28.7	2.5	10	LD (893.75), RA (1)	994
	M	67	81	169	3	30	2	14	LD (500), PR (1.57)	657
	M	64	94	190	3	30	2.5	22	LD (875), PR (0.52)	927
	M	73	92	172	12	28.3	2.5	27	EN (800), LD (706.25), RA (1), RT (4)	1159
	W	62	60	157	5	30	1.5	9	PR (0.52), RA (1)	152
	W	70	60	160	10	30	2	23	EN (200), LD (137.5), PR (1.05), RT (8)	528
	M	75	80	170	10	27.4	2.5	24	EN (600), LD (375), RO (16)	819
	W	66	65	168	2	27.9	2.5	41	RA (1), RO (14)	380
	W	71	50	147	1	27.7	2.5	17	RA (1), RT (6)	280
	W	64	75	160	5	30	2.5	24	LD (125), PR (3.15), RA (1)	540
	M	72	99	180	4	27.4	2	24	EN (600), LD (281.25), RT (6)	554
Mean	69.5	73.1	166.9	6.9	27.8	2.3	20.1			728.7
SD	5.5	15.6	11.2	4.0	2.3	0.3	8.0			390.7
Student's *t*-test	0.56	0.58	0.69	0.92						0.38
Mann–Whitney's *U* test					0.89	0.33	0.28			

SD: standard deviation; M: man; W: woman; MMSE: Mini-Mental State Examination; UPDRS: Unified Parkinson's Disease Rating Scale; AM: amantadine; EN: entacapone; LD: levodopa; PR: pramipexole; RO: ropinirole; RA: rasagiline; RT: rotigotine; LED: levodopa equivalent dose [98].

**Table 2:** Balance exercises administered to the PD-E group, based on the Otago Exercise Program [33] and guideline program for Parkinson's disease [38, 39].

Exercise	Description & dose	Progression
Tandem	Place a foot straight in front of the other, with the heel touching the toe. Keep your balance 30 s long, then reverse your feet. Repeat this exercise 3 times for each foot.	Difficulty was raised according to patient's skills. Starting with half-tandem (feet not near together), going on with tandem performed on different surfaces like foam and inclined ramp. Difficulty was further increased by keeping the eyes closed.

One leg stance	Look straight ahead. Keep your hands on your hips. Lift on your leg without touching or resting your raised leg upon other standing leg. Stay standing on one leg 30 s long, then switch between one foot and the other. Repeat this exercise 3 times for each foot.	Difficulty was raised according to patient's skills by utilizing different surfaces like foam or keeping one foot lifted up a step. Difficulty was further increased by keeping the eyes closed.

Inclined ramp	Stand upon the inclined ramp with toes toward the top. Place feet shoulder-width apart. Maintain the position 45 s long, then turn around in order to have the top by your side. Repeat for each side and one more time with the top behind.	Difficulty was raised according to patient's skills by closing up feet. Difficulty was further increased by keeping the eyes closed.

Stance	Place your feet together until almost touching, looking straight ahead. Be as stable and still as possible. Keep the position 60 s long and repeat 3 times each exercise.	Difficulty was raised according to patient's skills by performing exercises on foam surfaces, by closing up the feet or by keeping the eyes closed.

Compensatory stepping correction	Stand in front of the physiotherapist and lean on his hands. When support is released, make a step to maintain balance. In the same way, the physiotherapist can elicit backward stepping response and lateral stepping response.	Difficulty was increased by keeping the eyes closed.
